# Eastern equine encephalitis virus in mice I: clinical course and outcome are dependent on route of exposure

**DOI:** 10.1186/s12985-015-0386-1

**Published:** 2015-09-29

**Authors:** Shelley P. Honnold, Eric C. Mossel, Russell R. Bakken, Diana Fisher, Cathleen M. Lind, Jeffrey W. Cohen, Lori T. Eccleston, Kevin B. Spurgers, Rebecca Erwin-Cohen, Steven B. Bradfute, Radha K. Maheshwari, Pamela J. Glass

**Affiliations:** Virology Division, United States Army Medical Research Institute of Infectious Diseases, Fort Detrick, Frederick, MD 21702 USA; Integrated Toxicology Division, United States Army Medical Research Institute of Infectious Diseases, Fort Detrick, Frederick, MD 21702 USA; Present address: Department of Internal Medicine, Center for Global Health, University of New Mexico, Albuquerque, NM 87131 USA; Department of Pathology, Uniformed Services University of the Health Sciences, 4301 Jones Bridge Road, Bethesda, MD 20814 USA

**Keywords:** Alphavirus, Eastern equine encephalitis virus, Neuroinvasion, Mice

## Abstract

**Background:**

Eastern equine encephalitis virus (EEEV), an arbovirus, is an important human and veterinary pathogen belonging to one of seven antigenic complexes in the genus *Alphavirus*, family *Togaviridae*. EEEV is considered the most deadly of the mosquito-borne alphaviruses due to the high case fatality rate associated with clinical infections, reaching up to 75 % in humans and 90 % in horses. In patients that survive acute infection, neurologic sequelae are often devastating. Although natural infections are acquired by mosquito bite, EEEV is also highly infectious by aerosol. This fact, along with the relative ease of production and stability of this virus, has led it to being identified as a potential agent of bioterrorism.

**Methods:**

To characterize the clinical course and outcome of EEEV strain FL93-939 infection, we compared clinical parameters, cytokine expression, viremia, and viral titers in numerous tissues of mice exposed by various routes. Twelve-week-old female BALB/c mice were infected by the intranasal, aerosol, or subcutaneous route. Mice were monitored for clinical signs of disease and euthanized at specified time points (6 hpi through 8 dpi). Blood and tissues were harvested for cytokine analysis and/or viral titer determination.

**Results:**

Although all groups of animals exhibited similar clinical signs after inoculation, the onset and severity differed. The majority of those animals exposed by the aerosol route developed severe clinical signs by 4 dpi. Significant differences were also observed in the viral titers of target tissues, with virus being detected in the brain at 6 hpi in the aerosol study.

**Conclusion:**

The clinical course and outcome of EEEV infection in mice is dependent on route of exposure. Aerosol exposure to EEEV results in acute onset of clinical signs, rapid neuroinvasion, and 100 % mortality.

**Electronic supplementary material:**

The online version of this article (doi:10.1186/s12985-015-0386-1) contains supplementary material, which is available to authorized users.

## Background

Viruses of the genus A*lphavirus*, family *Togaviridae*, are significant human pathogens maintained by an enzootic cycle between mosquitoes and vertebrates. They can be generally divided by geography and pathology into the Old World alphaviruses such as Chikungunya, Ross River, and O’nyong-nyong viruses, which are usually associated with a mild to severe arthralgic disease in humans, and the New World alphaviruses such as western equine encephalitis virus (WEEV), Venezuelan equine encephalitis virus (VEEV), and eastern equine encephalitis virus (EEEV), which cause a mild to severe encephalitis in both humans and equids. Previously, EEEV was further divided into the highly pathogenic North American antigenic group (NA EEEV) and the three Central and South American antigenic groups, which only occasionally cause human disease (SA EEEV) [1, 2]. Recent changes in taxonomy identify NA EEEV strains as EEEV and SA EEEV are a new species designated Madariaga viruses (II-IV) [1]

Because of its high mortality (36-75 %) [3], lack of a licensed human vaccine and effective antiviral therapy, and its potential use as a biological weapon, EEEV is listed as a category B agent by the National Institute of Allergy and Infectious Diseases (NIAID). Therefore, research directed towards understanding the pathogenesis of EEEV, the difference in virulence between EEEV and Madariaga virus strains, and the development of a safe and effective vaccine and antiviral treatment for humans is essential. However, research has been hampered by the fact that mice develop age-dependent resistance to peripheral infection. Recently, EEEV, FL 93–939 was shown to be virulent in adult mice when administered peripherally; however there is limited information regarding the pathogenesis of this strain, with most studies focusing on the mechanism of decreased type I IFN induction in infected mice [4–6]. Characterizing the clinical course and outcome of EEEV strain FL93-939 in mice using various routes of infection is an important first step in understanding the pathogenesis of the NA EEEV strains, which is paramount when developing a vaccine and/or therapeutic to prevent the disease.

A comprehensive study was performed to determine and compare the clinical course of NA EEEV FL93-939 infection of mice following infection by the intranasal (IN), aerosol (AE), and subcutaneous (SC) routes. The AE route is the likely route of exposure to an EEEV bioweapon and one way by which laboratory workers may be accidentally exposed. However, many investigators do not have the equipment necessary to conduct AE studies and often use IN as a proxy exposure method. Identifying any differences in pathogenesis will be important for the proper interpretation of future studies utilizing either of these routes. The SC route of exposure is meant to mimic two unintentional exposure scenarios: laboratory exposure via contaminated needle puncture and natural infection from the bite of an infected mosquito, with the caveat that needle inoculation is not a perfect substitute for a mosquito bite [7].

Here the clinical course of disease, including clinical sign onset and duration, as well as complete blood count, serum analysis, and tissue distribution of virus over time following infection is presented. A subsequent manuscript will present tissue pathology associated with the various routes of exposure.

## Results

### Weight variance and clinical signs

**Fig. 1:**
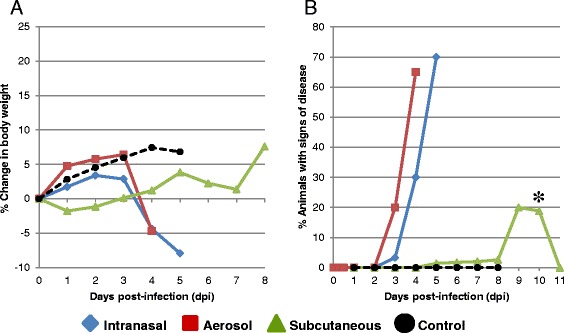
Change in mean body weight (**a**) and percent of BALB/c mice that either displayed clinical signs of disease or were moribund (**b**) after intranasal, aerosol, or subcutaneous infection with EEEV strain FL93-939. Mice were monitored daily after infection and percent change in weight was determined from the day of infection (day 0). Note that the aerosol study was terminated at 4 dpi because 90 % of the animals in the 5 dpi group had moderate to severe signs of clinical disease. (*) Three animals in the subcutaneous study from the 8 dpi group were euthanized prematurely due to the onset of severe clinical disease

The post-exposure weight variance and onset of clinical signs following IN and AE exposure were similar (Fig. 1). Both groups of mice continued to gain weight up to 2 dpi. However, the IN group began to lose weight by 3 dpi, while the AE group had minimal increases in weight over the same time period. For both groups, there was rapid weight loss from 3 dpi to 4 dpi and the IN group continued to lose weight through the end of the study, 5 dpi. The AE study was terminated on 4 dpi because 90 % of the animals that were scheduled to be euthanized at 5 dpi were showing moderate to severe signs of clinical disease. In both the IN and AE studies, mice began displaying clinical signs of disease (ruffled fur, lethargy, hunched posture) at 3 dpi and both the percentage of animals affected and the severity of clinical signs increased from 3 dpi to the study endpoints. More severe clinical signs of disease included weight loss, dehydration, head tilt, circling, head tremors, focal muscle twitching, lateral recumbency, and rarely seizures.

On the other hand, mice exposed by the SC route initially lost weight at 1 dpi but gradually gained weight over the following 5 days. Mice began losing weight after 5 dpi, which coincided with the onset of clinical signs in most animals. Three of the ten animals from the 8 dpi group were moribund and were euthanized on either 6 dpi or 7 dpi. The 7 remaining animals in the 8 dpi group were clinically normal and gaining weight at 8 dpi. The clinical signs of disease observed in the SC study were similar to those noted in the IN and AE studies but occurred in fewer animals and generally were not noted until 6 dpi. However, one animal in the 8 dpi group did show clinical signs of disease starting on 2 dpi. Disease progressed slowly in this animal until it was euthanized on 7 dpi due to severe clinical signs of disease.

### Temperature and activity

**Fig. 2:**
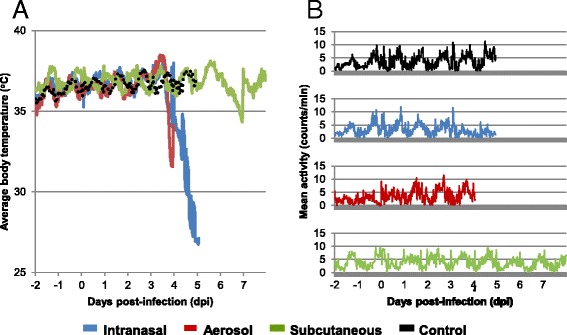
Change in average body temperature (**a**), and activity (**b**) after intranasal, aerosol, or subcutaneous infection with NA EEEV strain FL93-939. Body temperatures and activity were recorded for a 20 sec period every 30 min via an intraperitoneal telemetry device before and after infection with EEEV strain FL93-939. Values represent the average within the cohort (intranasal n = 5; aerosol n = 5; subcutaneous n = 9; control n = 15). Note that the aerosol study was terminated at 4 dpi because 90 % of the animals in the 5dpi group were showing moderate to severe signs of clinical disease

Temperature and activity data were collected from EEEV infected and uninfected BALB/c mice to evaluate differences between various routes of infection and age-matched controls. The average daily temperature range was 35.2 - 37.8 °C. The mean temperature profile for each group is shown in Fig. 2a. The diurnal temperature pattern in IN and AE EEEV infected mice began to deviate from the pattern observed in control mice approximately 3–4 dpi, which coincided with onset of clinical signs. In SC infected animals, the diurnal temperature pattern was unchanged until 6–7 dpi, which coincided with the time at which the highest percentage of animals displayed clinical signs. The fever, detected by telemetry at 3 dpi for the IN and AE studies and 5 dpi in the SC study (Table 1), was the first indication that telemetric analysis coincided with the daily cage-side observations in detecting onset of disease. However, when comparing infected to uninfected mice within each study, only the infected animals in the AE and SC studies showed a significant difference in the duration and total fever hours relative to uninfected controls (p < 0.05), which may be a result of the small group numbers. In the AE study, 4 of 5 animals had a fever of significant duration; however, in the IN study only 3 of 5 animals had a fever for 2 or more consecutive time points. Nevertheless, the p-value in the IN study was just slightly above 0.05 for both duration and total fever hours (p = 0.08 for duration and p = 0.09 for total fever hours).

**Table 1:** Summary of fever data for IN, AE, and SC studies

	Group	Onset^a^	ΔTmax, °C	Duration, h^b^	Fever hours^c^	Average elevation^d^
IN	Infected	3.0	1.9	11.3	28.2	1.9
	Controls	--	1.1	0.6	1.1	1.1
	p-value	--	0.2972	0.0822	0.0913	0.3166
AE	Infected	3.0	2.3	11.6	26.5	1.8
	Controls	--	0.5	0.4	0.9	0.4
	p-value	--	0.0405	0.0302	0.0350	0.0670
SC	Infected	5.0	1.8	5.9	12.9	1.6
	Controls	--	0.7	0.2	0.4	0.7
	p-value	--	0.1000	0.0436	0.0485	0.1144

ΔT_max_ = the maximum change in temperature

^a^Defined as the first day with >8 hours of significant temperature elevation (as determined by ARIMA modeling)

^b^Calculated as the number of hours of significant temperature elevation

^c^Calculated as the sum of the significant temperature elevations

^d^Calculated by dividing fever hours by fever duration in hours

Distinct diurnal patterns in activity were recorded over a 24 hr period in all groups, with activity generally peaking during nocturnal time points and reaching daily lows in the morning hours (Fig. 2b). When compared to control mice, mice in both the IN and AE studies deviated from the pattern observed in control mice between 3–5 dpi, again coinciding with the onset of clinical signs.

### CBC analysis

**Fig. 3:**
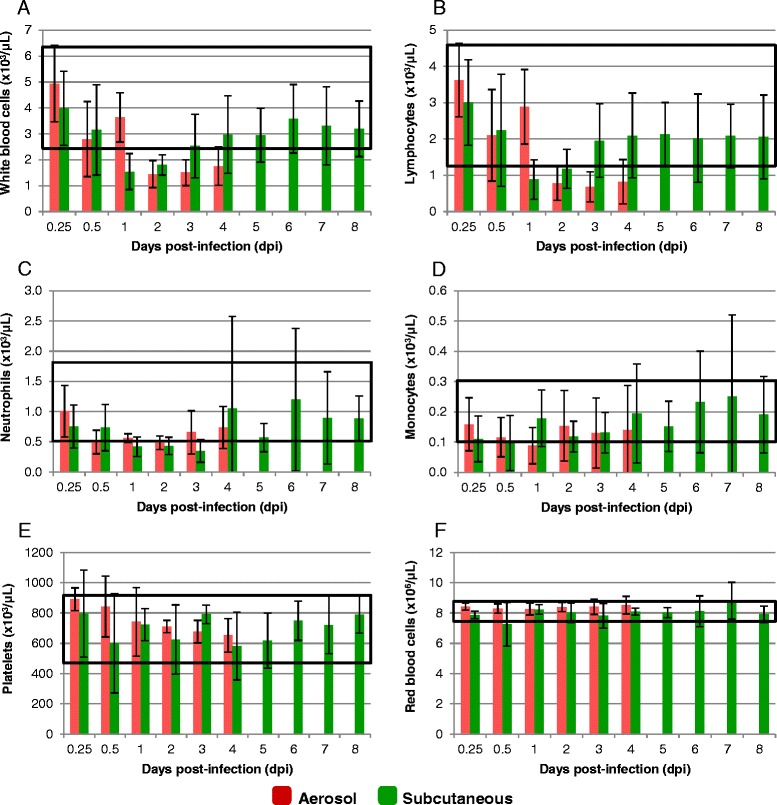
Complete blood counts in mice exposed to EEEV strain FL93-939 either by the aerosol or subcutaneous routes. Numbers shown are the mean ± standard deviation of total white blood cells (**a**), lymphocytes (**b**), neutrophils (**c**), monocytes (**d**), platelets (**e**), and red blood cells (**f**), at each time point (n = 10 per time point per route). Normal ranges (black boxes) were determined from control animals (n = 20) combined from both routes of exposure (white blood cells = 2.4-6.3 x 10^3^/μL, neutrophils = 0.5-1.8 x 10^3^/μL, lymphocytes = 1.2-4.6 x 10^3^/μL, monocytes = 0.1-0.3 x 10^3^/μL, red blood cells = 7.6-8.7 x 10^6^/μL, and platelets = 458.9-914.6 x 10^3^/μL). Note that the aerosol study was terminated at 4 dpi because 90 % of the animals in the 5dpi group were showing moderate to severe signs of clinical disease

When an adequate volume of blood was collected at euthanasia, a complete blood count was performed on individual animals from the AE and SC studies. Group mean results are shown in Fig. 3. Significant variation among individuals was observed, which resulted in large standard deviations in some groups. However, in AE infected animals, there was a general decrease in the total number of white blood cells (WBC) (leukopenia) by 2 dpi, with the group mean value dropping below the normal (control group) range and remaining low through the end of the study. This decrease in WBCs was characterized by a decrease in lymphocytes (lymphopenia), while the number of neutrophils and monocytes (Fig. 3a-d), as well as the number of eosinophils and basophils (data not shown), remained within the normal range. Significant differences (p < 0.05) were observed in total WBC and number of lymphocytes between infected and uninfected animals from 12 hpi through the end of the study (Table 2). Interestingly, while the mean total number of neutrophils in infected animals never fell below the normal range at any time point, there were significant differences in the total number of neutrophils present in infected and uninfected animals at every time point. There were also significant differences in the number of platelets at 0.25 dpi and 0.5 dpi hpi. A leukopenia was also observed in SC infected animals; however, this occurred earlier, at 1 dpi, and when evaluating group means, was characterized by both a lymphopenia as well as a neutropenia. These values remained low through 2 dpi for lymphocytes and 3 dpi for neutrophils, after which time all mean values returned to the normal range for the remainder of the study. No significant differences between SC infected and uninfected animals were observed at any of the time points. The AE and SC group mean values for red blood cells remained within the normal range for the duration of the study.

**Table 2:** P-values for the pairwise comparisons of mean blood parameters between animals infected by the AE or SC route and uninfected animals (n = 10)

		Days post-exposure (dpi)									
Study	Blood Parameter	0.25	0.5	1	2	3	4	5	6	7	8
AE	WBC #	0.0563	<0.0001	<0.0001	<0.0001	<0.0001	<0.0001	<0.0001	--	--	--
	Neutrophil #	0.0366	<0.0001	<0.0001	<0.0001	0.0001	0.0002	0.0210	--	--	--
	Lymphocyte #	0.1292	<0.0001	0.0038	<0.0001	<0.0001	<0.0001	<0.0001	--	--	--
	Monocyte #	1	0.7840	0.2948	1	1	0.2948	1	--	--	--
	Eosinophil #	1	1	1	1	0.7126	1	1	--	--	--
	Basophil #	0.0086	0.9014	0.8748	0.0473	0.1029	0.2591	0.7711	--	--	--
	Hematocrit	0.1232	0.6098	0.7575	0.7030	0.6373	0.7575	0.0297	--	--	--
	Platelet #	0.0016	0.0058	0.2013	0.4579	0.6875	0.8702	0.8702	--	--	--
SC	WBC #	0.5959	1	0.1037	0.3519	1	1	1	1	1	1
	Neutrophil #	1	1	0.9472	0.9975	0.7343	1	1	1	1	1
	Lymphocyte #	0.0557	1	0.6456	1	1	1	1	1	1	1
	Monocyte #	0.2604	0.2320	1	0.3549	0.4931	1	0.8363	1	1	1
	Eosinophil #	1	1	1	1	1	1	1	0.1933	1	1
	Basophil #	1	1	0.5224	1	1	1	1	1	1	0.9507
	Hematocrit	1	0.0886	1	1	1	1	1	1	1	0
	Platelet #	1	0.7499	1	0.9118	1	0.4313	0.8769	1	1	0

### Cytokine analysis

**Fig. 4:**
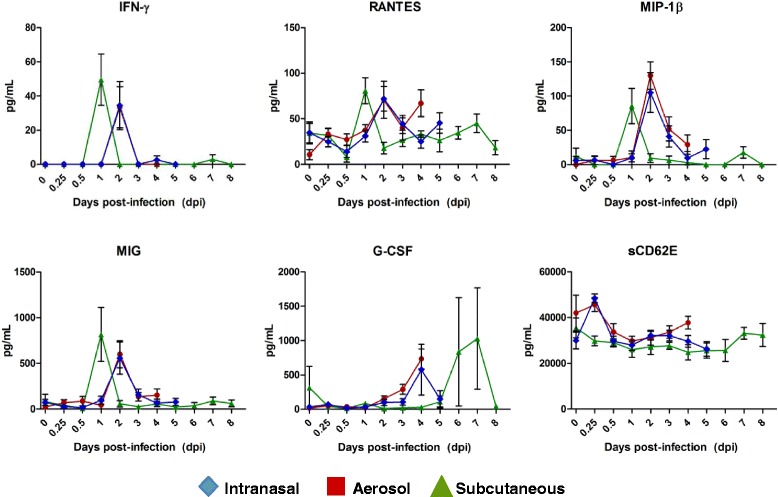
Serum analysis of soluble proteins in mice infected with EEEV strain FL93-939 by the intranasal, aerosol, or subcutaneous route. Infected mice were euthanized at specified time points. Soluble proteins in the serum were determined using BD Cytometric Bead Array mouse soluble protein flex sets and analyzed using FCAP Array software. Values represent the group mean at each time point (n = 6), bars represent the standard deviation. Mean baseline values (0 dpi) were determined from uninfected controls (n = 6)

To characterize and compare route-specific induction of inflammatory and immunomodulatory cytokines and chemokines, a subset of serum samples were analyzed from the IN, AE, and SC studies for the presence of 25 soluble proteins (sCD62E, sCD62L, G-CSF, GM-CSF, IFN-γ, IL-1α, IL-1β, IL-2, I L-3, IL-4, IL-5, IL-6, IL-9, IL-10, IL-12/IL23p40, IL-13, IL-17A, IL-21, KC/CXCL1, MCP-1/CCL2, MIG.CXCL9, MIP-1α/CCL3, MIP-1β/CCL4, RANTES/CCL5, and TNF). There were no significant differences in any of the 25 cytokines and chemokines tested between aerosol and intranasal infection at any time point. However, there were several differences between subcutaneous and intranasal or aerosol infections (Fig. 4). Subcutaneous infection resulted in an increase of IFN-γ, MIP-1β, RANTES, and MIG at 1 dpi compared to aerosol or intranasal infection, followed by a return to baseline at 2 dpi. These same cyto/chemokines were increased in aerosol and intranasal infection at 2 dpi relative to subcutaneous infection. Infection by all three routes generated a late increase in G-CSF levels: 4 dpi following IN or AE infection and 6–7 dpi after SC infection.

### Virus titrations

Viral titers in the serum, bronchoalveolar lavage (BAL), nasopharyngeal flush (NF), and several tissues were determined by standard plaque assay. Large differences in viral load were detected in most samples based on the route of infection. However, substantial variability was noted between animals in any one group. For this reason, the data shown is for both individual animals as well as group mean titers.

**Fig. 5:**
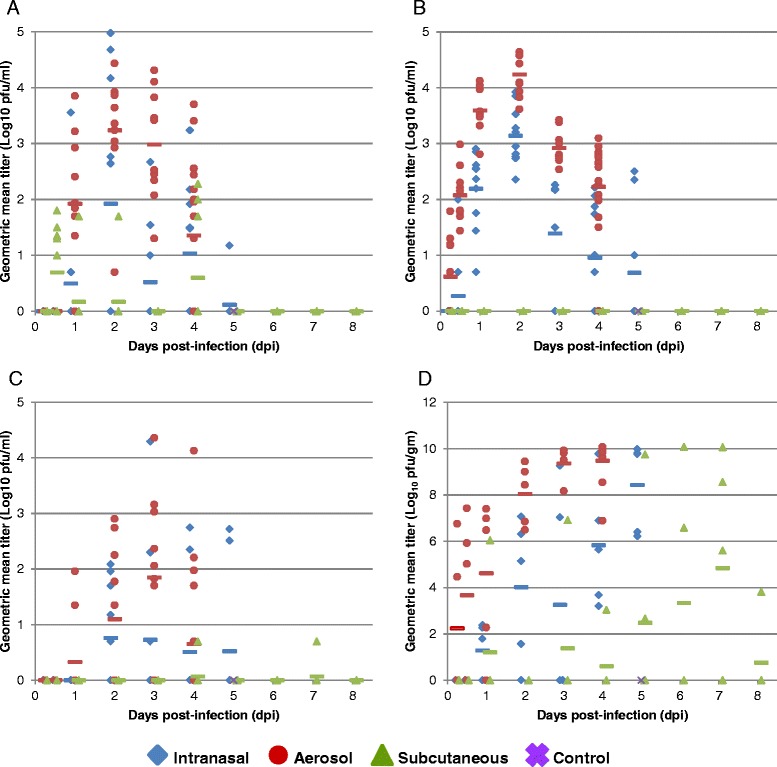
Distribution of EEEV FL93-939 in fluids and tissues of BALB/c mice exposed by various routes. Shown are the geometric mean virus titer of individual animals in the serum (**a**), bronchoalveolar lavage (BAL) (**b**), nasopharyngeal flush (NF) (C) and brain (D) (n = 5/time point). Symbols represent individual animals with values calculated from the geometric mean titer of all dilutions which had at least one visible pfu by standard plaque assay. The mean for the group is shown in the colored dashed line. The limit of detection of the assay is 5 pfu/ml fluid or tissue homogenate supernatant

Viremia was first detected in the SC study at 12 hpi, while viremia was not detected until 1 dpi in either the IN or AE studies (Fig. 5a). This was likely due to the route of infection and rapid viral replication at the inoculation site. However, viremia remained low in the SC study and was not detected after 4 dpi. In contrast, while viremia appeared at 1 dpi in both the IN and AE studies, it peaked at 2 dpi, was substantially higher than the SC study, and was present until the study endpoint.

As expected, no virus was detected in the BAL or NF during the early time points in the SC study (Figs. 5b and c). Substantially more virus was present in the BAL at 6 hpi and 12 hpi in the AE study as compared to the IN study, and virus levels remained higher throughout the study. Virus was not present in the NF until 1 dpi in the AE study and 2 dpi in the IN study. Similar to the titers in the BAL, those in the NF were higher in AE study throughout all time points, although the overall titers were lower and the differences less distinct. These observations were likely due to the difference in delivery method. The collision nebulizer used for the AE study is designed to generate small 1 μm particles, many of which are small enough to be delivered into the terminal bronchioles, while some particles are likely to remain in direct contact with olfactory nasal epithelium, allowing for attachment, entry, and replication. Virus in the IN study was delivered in liquid form, 10 μL per nostril, some may have remained outside the nostril and some may have been swallowed or inhaled.

**Fig. 6:**
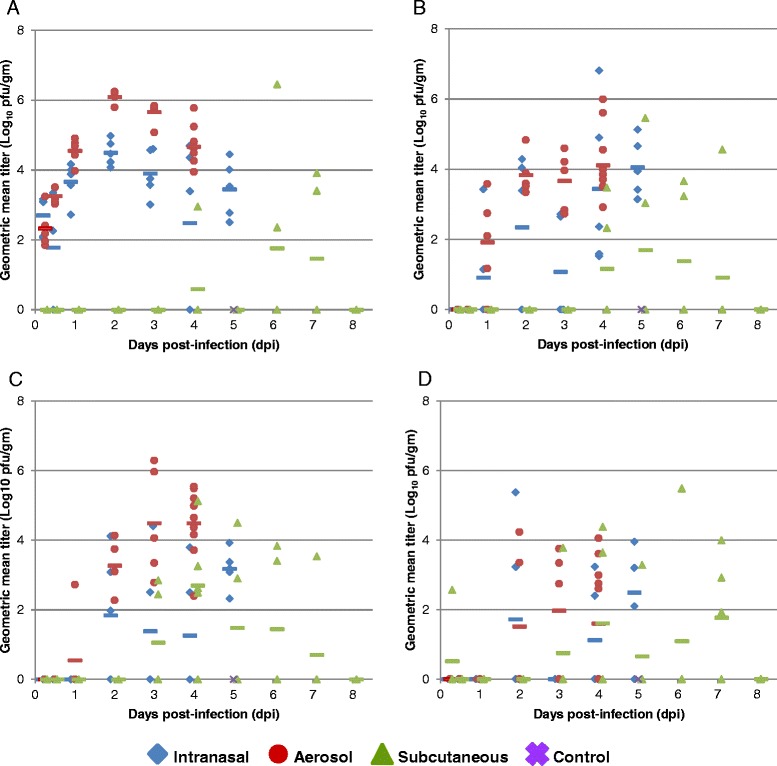
Distribution of EEEV FL93-939 in tissues of BALB/c mice exposed by various routes. Shown are the geometric mean virus titer of individual animals in the lung (**a**), submandibular salivary gland and lymph node (**b**), spleen (**c**), and mesenteric lymph node (**d**) (n = 5/time point). Symbols represent individual animals with values calculated from the geometric mean titer of all dilutions which had at least one visible pfu by standard plaque assay. The mean for the group is shown in the colored dashed line. The limit of detection of the assay is 5 pfu/ml tissue homogenate supernatant.

Virus titers in the tissues followed similar trends. Although the mice in the IN and AE studies received similar LD_50_ doses of virus, virus was present in the brain 6 hpi in the AE study, but did not appear until 1 dpi in the IN study (Fig. 5d). Virus titers in the brain continued to increase in both groups, with mice of the AE group consistently having higher titers than mice in the IN group at all time points, peaking with very high titers at 4 dpi in the AE study and 5 dpi in the IN study. In the SC study, virus was first detected in one animal in the brain at 1 dpi, similar to the IN study; however, titers remained lower in the SC study, as compared to the AE and IN studies, for all time points, with virus titer peaking later, at 7 dpi. Virus titers in the lung (Fig. 6a) in the IN and AE studies were similar at 6 hpi, and in both studies continued to rise to fairly high titers through 2 dpi; however, the titers in the AE study were significantly higher than those in the IN study from 2–4 dpi. Virus was not present in the lung in the SC study until 4 dpi and titers remained low even at 6–7 dpi. The mandibular salivary gland and lymph nodes were collected and analyzed together due to their intimate association anatomically. The mandibular lymph nodes, also known as the mandibular and accessory mandibular lymph node, submandibular lymph nodes, or superficial cervical lymph nodes [8], are a small group of lymph nodes that drain the structures of the muzzle [9] and were therefore of interest in the IN and AE studies. As might be expected, virus was present at low levels in the mandibular lymph nodes in both the IN and AE studies at 1 dpi (Fig. 6b) and titers slowly increased throughout the remaining time points, peaking at 5 dpi and 4 dpi, respectively. In the SC study, virus was not present in the mandibular lymph nodes until 4 dpi and remained at relatively low levels through 7 dpi. Virus at this site could be the result of drainage from the nasal cavity or seeding from viremia. Virus titers in the spleen (Fig. 6c) followed similar trends, with virus first appearing in the spleen at 1 dpi in the AE study and at 2 dpi in the IN study; titers increased throughout the study and peaked at 4 dpi and 5 dpi, respectively. In the SC study, virus appeared at 3 dpi, peaked at 4 dpi and slowly decreased over the remaining time points. The mesenteric lymph node, which drains the digestive tract, is a distant lymph node for all routes of infection, and virus present in this lymph node may be indicative of distant viral spread by either blood or lymph. The virus titers in this tissue were low for all routes of infection and peaked at 3 dpi in the AE study, 5 dpi in the IN study, and 7 dpi in the SC study (Fig. 6d). Mean group virus titers of the remaining tissues (liver, heart, kidneys, adrenal glands, and pancreas) evaluated in all three studies are shown in Additional file 1: Figure S1. No virus was present in any of these tissues until 1 dpi and virus titers remained very low throughout most time points. The slight increase in titer in the heart and kidneys could be due to poor perfusion of these vascular organs with PBS prior to tissue collection. Overall, the differences in virus titer in the various tissues are likely due to the variation in delivery method and virus dose. In the IN and AE studies, in which mice received approximately 100LD_50_, virus was present early on and at high titer in the serum, BAL, brain, and lung. The viral titer in the remaining tissues was lower and peaked later in the time course. While in the SC study, in which mice received approximately 30LD_50_, the viral titer in tissues was generally lower and peaked later in the course of disease.

**Fig. 7:**
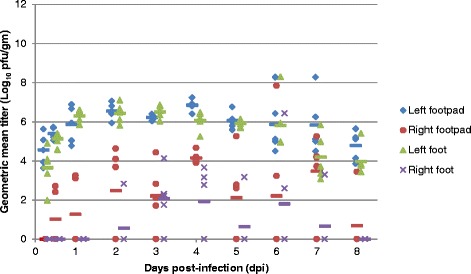
Geometric mean virus titer in the left and right footpad and foot of individual BALB/c mice infected SC with EEEV strain FL93-939 in the left rear footpad (n = 5). Viral titers of tissue homogenate supernatants were determined by standard plaque assay. Symbols represent individual animals with values calculated from the geometric mean titer of all dilutions which had at least one visible pfu. The mean for the group is shown in the colored dashed line. The limit of detection of the assay is 5 pfu/ml tissue homogenate supernatant

To more accurately trace the path of the virus in the SC study, mice were inoculated in the left rear footpad and subsequent samples were collected from the left and right footpad, foot, gastrocnemius muscle, and popliteal lymph node for viral titer. As expected, the viral titer was high in the left footpad and left foot early in infection (6 hpi) and remained high throughout all time points, whereas virus appeared at low levels in the right footpad and right foot after 12 hpi and 2 dpi and peaked at 4 dpi and 3 dpi respectively (Fig. 7). There was significant viral replication at the site of inoculation (left footpad and foot) as the mice received approximately 1000 pfu/footpad and the titers reached 10^6^ pfu/gm by 1 dpi.

**Fig. 8:**
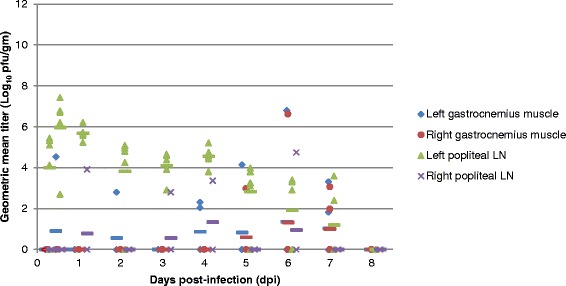
Geometric mean virus titer in the left and right gastrocnemius muscle and popliteal lymph node of individual BALB/c mice infected SC with EEEV strain FL93-939 in the left rear footpad (n = 5). Viral titers of tissue homogenate supernatants were determined by standard plaque assay. Symbols represent individual animals with values calculated from the geometric mean titer of all dilutions which had at least one visible pfu. The mean for the group is shown in the colored dashed line. The limit of detection of the assay is 5 pfu/ml tissue homogenate supernatant

Virus titers were determined for the left and right gastrocnemius muscle (calf muscle), a large muscle group in the lower leg, as it has been previously reported that EEEV replicates in skeletal muscle [10]. The mean viral titer of left gastrocnemius muscle remained low throughout the study and virus did not appear in the right gastrocnemius muscle until 5 dpi (Fig. 8). Virus titers were also determined for the left and right popliteal lymph nodes, the draining lymph nodes of the inoculation site. The mean virus titer of the left popliteal lymph node rose rapidly post-inoculation and remained at high levels until 6 dpi. While virus was detected in the right popliteal lymph node 1 dpi, viral titers were more sporadic and generally remained low throughout the study.

## Discussion

To better understand the pathogenesis of EEEV strain FL93-939 in mice, several studies were conducted to evaluate the differences between 3 routes of infection: intranasal, aerosol, and subcutaneous. Although the intranasal and aerosol routes were similar, there were important differences noted. While animals in both groups lost weight between 3–4 dpi, those animals in the AE study displayed clinical signs of disease at 3 dpi compared to 4 dpi in the IN study. The clinical signs were so severe in the majority of animals in the AE study at 4 dpi that the study was terminated early. Clinical signs of disease did not appear until 5 dpi in the SC study. Interestingly though, the absolute dose given per mouse was similar between the IN and SC routes, 1300 pfu and 1000 pfu respectively, yet the outcome was drastically different with regard to clinical disease onset and viral tissue distribution and titers. This is likely due to differences in inoculation site, the resultant immune response, and the relative lethal dose given; 100LD_50_ in the IN study compared to 30LD_50_ in the SQ study. The clinical signs noted in the SC study were similar yet less severe than previously reported [10]; however, mice in the Vogel et al. study had severe clinical signs by 4 dpi. The difference in onset of clinical signs and severity noted in the Vogel study may be attributed to the virus strain (FL91–4679), higher inoculum (10^5^ pfu), the mouse strain (C57/BL6), or the age of the mice (5-weeks) used in that study, or most likely a combination of these factors. The clinical findings in our SC study more closely paralleled those noted recently by Gardner et al. [11] in which they did not observe signs of disease until 6 dpi following a subcutaneous exposure and weight loss was minimal over the course of the study.

Telemetry was used previously to study non-human primates infected with EEEV by the aerosol route [12]. However, telemetry had not been used previously to study EEEV infection in mice, or to compare routes of infection. As in the non-human primate study, telemetry proved to be a useful tool in determining onset of clinical disease in mice. Fever was detected at 3 dpi in both the IN and AE studies, which coincided with the onset of mild signs (ruffled fur). Additionally, obvious changes in diurnal patterns in both temperature and activity coincided with the onset of more severe clinical signs of disease. This study highlighted the importance of monitoring such parameters and revealed that the clinical signs in mice, similar to those seen in humans, included fever prior to or at the onset of more obvious clinical signs of disease, such as ruffled fur and lethargy.

A complete blood count was performed on animals from the AE and SC studies; however, the results were not specific or predictive of outcome. Most animals had a leukopenia characterized by a lymphopenia at 2–3 dpi, which can be indicative of a viral infection. These results were similar to those reported by Adams et al. [13] in which marmosets infected with EEEV had a leukopenia 1 dpi, but a concomitant decrease in neutrophils, lymphocytes and monocytes. In the marmosets, this blood profile rapidly changed to a leukocytosis, characterized by a neutrophilia, lymphocytosis and a monocytosis by 3–4 dpi. While there was a rise in leukocytes in our SC study after 3 dpi, the number of leukocytes remained within the normal range for the study duration. It is difficult to compare CBC results in research models to that observed in human cases, since the infectious dose and time from inoculation to presentation in humans is typically not known.

Cytokine analysis in EEEV infected research models has not been reported. While there were several pro-inflammatory cytokines and chemokines that were elevated to statistically significant levels relative to saline-treated control animals, most of the elevated cyto/chemokine levels were only slightly increased from baseline. However, temporal comparison of cytokine levels between various routes of infection revealed some interesting results. That no significant differences were observed among any of the 25 cytokines and chemokines tested between aerosol and intranasal infection at any time point suggests these routes of infection induced very similar immune responses. However, several differences were noted between subcutaneous and intranasal or aerosol infections. IFN-γ, MIP-1β (a chemoattractant for macrophages and NK cells), RANTES (a chemokine for T-cells), and MIG (a T-cell attractant and activator) were increased one day following subcutaneous infection relative to aerosol or intranasal infection, followed by a return to baseline one day later when the same cyto/chemokines become increased in aerosol or intranasal infection. sCD62E, a soluble analogue of the E-selectin cell adhesion molecule, may also have been increased in both IN and AE infected animals early in infection. However, the relatively high level observed in AE-infected day 0 controls prevents a conclusive association. Overall, these data suggest a mild induction of certain pro-inflammatory cytokines and chemokines after infection. It is possible that the earlier spike in IFN-γ, MIP-1β, RANTES, and MIG levels after subcutaneous infection contributed to the decreased virulence seen in these mice compared to intranasal or aerosol infection. It is noteworthy that this study focused on serum cytokine and chemokine levels, which may reflect proteins produced by a number of sources, including circulating immune cells and various target tissues. Future studies should focus on determining tissue cytokine levels to further elucidate their source and potential role in infection and disease control or exacerbation.

In these studies, virus titration of tissue homogenates was used to characterize the temporal progression of EEEV FL93-939 from the site of exposure/inoculation to the CNS of infected mice. In IN infected animals, virus was first detected in the blood and the brain homogenate at 1 dpi. By 2 dpi, viremia peaked and the virus titer in the brain rapidly increased. These data could be suggestive of a vascular route of neuroinvasion or invasion of the CNS via the olfactory route as observed in guinea pigs exposed to aerosolized EEEV [14]. However, further studies using IHC strongly indicate EEEV neuroinvasion following IN exposure follows the olfactory route [15].

The results for the AE study were very similar to the results of the IN study, with the important exception that virus was detected in the brain by titer at only 6 hpi following aerosol exposure. The aerosol delivery method likely allowed more virus contact with olfactory neurons, thus facilitating the earlier viral invasion of the olfactory bulb and subsequent transport to the olfactory tract and beyond [15]. These results support the clinical findings of more rapid and severe disease onset in this study.

The results of the SC study were less clear. Despite convincing evidence of virus replication near the site of inoculation and viremia in 30-40 % of animals at the early time points, viral infection of the brain was not consistently observed. While virus was first detected in the brain by plaque assay at 1 dpi (1 of 5 animals, 20 %), virus was only detected in the brain of 10 of 60 (17 %) of animals from 3–8 dpi. This may be due to variation in LD_50_ dose, immune response, or route of neuroinvasion.

In humans, the incubation period following natural EEEV infection is short, usually 4–10 days. Systemic infection is often characterized by abrupt onset of chills and fever followed by malaise, arthralgia, and myalgia. Typically these are difficult parameters to measure in animals; however, telemetry allowed for monitoring of temperature and activity and fever and decreased activity (lethargy or malaise) were noted in many infected animals. Clinical signs of encephalitis in humans include abrupt onset of severe fever, intense headache, irritability, restlessness, drowsiness, anorexia, nausea, vomiting, diarrhea, cyanosis, convulsions, and coma. Again, while most of these clinical signs cannot be evaluated in mice, marked lethargy and tremors were noted in some infected animals. In humans, death usually occurs within 2–14 days after the onset of clinical signs [16]. In these studies, mice generally were moribund or succumbed to infection within 2–4 days following the onset of clinical signs.

## Conclusion

Together with the follow on report describing the histopathology [15], this study completes a comprehensive characterization of the infection of BALB/c mice with EEEV strain FL93-939 by the aerosol, intranasal, and subcutaneous routes of exposure. These studies confirm that clinical course, pathogenesis, and outcome of infection with EEEV are dependent on route of exposure in mice. Understanding the differences in disease course commenced via different exposure routes is essential for the development of effective and robust vaccines and antiviral drugs.

## Methods

### Mice

Specific pathogen free 8–10 week-old female BALB/c mice (NCI, Frederick, MD) were housed in cages equipped with microisolators and were provided food and water *ad libitum* throughout the study. The room temperature was maintained at 23 ± 1 °C and periods of light and dark were on a 12 h cycle. Mice were acclimated for 1 week after which 10 animals in each study were surgically implanted with intraperitoneal telemetry devices (TA-F20, Data Sciences International, St. Paul, MN) to monitor body temperature and activity. Animals received 1 week post-operative recovery, thus weighed approximately 20 gm, and were 10–13 weeks old at the time of exposure. For the portions of the study involving live EEEV, mice were housed in a biosafety level 3 (BSL-3) facility. Human end points were used during all mouse studies. Research was conducted under an IACUC approved protocol in compliance with the Animal Welfare Act, Public Health Service Policy, and other Federal statutes and regulations relating to animals and experiments involving animals. The facility where the research was conducted is accredited by the Association for the Assessment and Accreditation of Laboratory Animal Care International and adheres to principles stated in the Guide for the Care and Use of Laboratory Animals, National Research Council, 2011.

### Virus

EEEV strain FL93-939 was obtained from Dr. Scott Weaver, UTMB, Galveston, TX. A sucrose-purified working stock was prepared from seed stock (P1) through an additional passage (P2) in Vero cells. Virus titer was determined by standard plaque assay on Vero cell monolayers. Virus was aliquoted and frozen at −70 to −80 °C prior to use. Challenge virus was diluted in either Eagle’s minimum essential medium (EMEM) (Cellgro, Mediatech, Inc., Manassas, VA) or sterilized phosphate buffered saline (PBS) (GIBCO Invitrogen Corp., Grand Island, NY).

### Experimental design

Groups of 10 mice were exposed to approximately 100LD_50_ of EEEV strain FL93-939 by either the intranasal, aerosol or subcutaneous route. For the intranasal route of exposure, virus dose was prepared in a 20 μL volume in sterilized PBS. Control mice received only sterilized PBS. Mice were briefly anesthetized with isoflurane using the IMPAC^6^ (VetEquip, Inc., Pleasanton, CA) and given 10 μL of challenge virus per nostril. For the aerosol route of exposure, virus dose was prepared in a 10 ml volume in EMEM. Control mice were exposed to diluent only. Aerosol exposures were conducted in a whole-body bioaerosol exposure system. A Collison nebulizer (BGI, Inc., Waltham, MA) was used to generate small (1 μm mass median aerodynamic diameter) diameter particles for each acute 10 min exposure. Briefly, mice were placed in wire cages, which were then placed into a chamber where they were exposed to aerosolized virus for 10 min. ‘Presented’ dose was estimated by calculating the respiratory minute volume (V_m_) using Guyton’s formula [17], expressed as V_m_ = 2.10 x W_b_^0.75^ where W_b_ = body weight (gm) based on the average group weights the day of exposure. The presented dose was then calculated by multiplying the estimated total volume (V_t_) of experimental atmosphere inhaled by each animal (V_t_ = V_m_ x length of exposure) by the empirically determined exposure concentration (C_e_) (‘presented dose’ = C_e_ x V_t_). Exposure concentration, expressed in plaque-forming units (PFU)/L, was determined by isokinetic sampling of the chamber with an all-glass impinger (AGI) (Ace Glass, Vineland, NJ). Samples were titrated by standard plaque assay on Vero cell monolayers [14]. For the subcutaneous route of exposure, virus dose was prepared in a 10 μL volume in EMEM. Mice were inoculated in the left foot pad in order to track viral replication in the surrounding tissue and draining lymph node (popliteal lymph node). Control mice received diluent only. Challenge virus preparations were back-titrated by standard plaque assay using Vero cells. Mice from the intranasal and aerosol studies were euthanized at pre-determined time points: 6, 12, 24, 48, 72, 96, and 120 hours post-infection (hpi). In addition to the previous listed time points, mice in the subcutaneous study were also euthanized at 144, 168, and 192 hpi. At the time of euthanasia, mice were anesthetized with mouse K-A-X (50 mg ketamine (Fort Dodge Animal Health, Fort Dodge, IA), 0.5 mg acepromazine (Boehringer Ingelheim, Ridgefield, CT), and 5.5 mg xylazine (Lloyd Laboratories, Walnut, CA)) given intraperitoneally at a dose of 0.2 ml per 20 gm. Mice were euthanized by exsanguination via cardiac puncture and whole blood samples were collected for CBC analysis, while serum samples were collected for viral titer and cytokine analysis. Five mice from each time point were perfused with PBS and tissues were individually collected and frozen for viral titer analysis.

### Acquisition and analysis of telemetry data

All telemetry data was collected using the DSI DataQuest ARTM™ software. The system was programmed to sample body temperature and physical activity for a 20 sec period every 30 min. Baseline data was collected for 2 days. Data collection continued until euthanasia or the end of the study. Pre-exposure temperature data was used to develop a baseline period to fit an autoregressive integrated moving average (ARIMA) model. Forecasted values for the post-exposure period were based on the baseline extrapolated forward in time using SAS ETS (v. 9.2). Residual changes were determined by subtracting the predicted value from the actual value recorded for each time point. For temperature, residual changes greater than two standard deviations were used to compute fever duration (number of hours of significant temperature elevation), fever hours (sum of the significant temperature elevations), and average fever elevation (fever hours divided by fever duration in hours). Only time periods consisting of two or more consecutive time points of elevated temperature were used in the analysis.

### CBC analysis

A complete blood count (CBC) was determined on whole blood samples collected at the time of euthanasia. Samples were run on an Abbott CELL-DYN 3700 with veterinary package (Abbott Laboratories, Abbott Park, IL) on the same day as collection. This instrument produces a differential white blood cell count based on size, internal granularity, and nuclear content using optical and impedance technology. T-tests with stepdown Bonferroni adjustment were used to compare mean levels of blood parameters between infected groups and uninfected control groups at various time points.

### Cytokine analysis

Cytokines/chemokines were measured on selected serum samples using BD™ Cytometric Bead Array mouse soluble protein flex sets (BD Biosciences, San Jose, CA) read on a BD FACSCanto II flow cytometer as per manufacturer’s instructions. Twenty-five soluble proteins were measured (sCD62E, sCD62L, G-CSF, GM-CSF, IFN-γ, IL-1α, IL-1β, IL-2, I L-3, IL-4, IL-5, IL-6, IL-9, IL-10, IL-12/IL23p40, IL-13, IL-17A, IL-21, KC/CXCL1, MCP-1/CCL2, MIG.CXCL9, MIP-1α/CCL3, MIP-1β/CCL4, RANTES/CCL5, and TNF) and results were analyzed using FCAP Array software (BD Biosciences).

### Mucosal secretions

A bronchoalveolar lavage (BAL) and nasopharyngeal flush (NF) were performed using 0.5 ml of sterile PBS for each. Briefly, under deep anesthesia, the trachea was exposed and an 18G needle was inserted toward the lower or upper respiratory tract, respectively. PBS was flushed into the lungs and aspirated for BAL or through the nares and/or oropharynx for nasopharyngeal flushes. Mice were then euthanized by exsanguination via cardiac puncture.

### Virus titrations

For determination of EEEV titers, tissue samples were homogenized using a mini-bead beater and 1–2 stainless steel beads (3.2 mm diameter) (BioSpec Products, Inc., Bartlesville, OK) and 500 μL of complete medium. Homogenized samples were centrifuged for 10 min at 10,000 rpm in a table-top centrifuge and supernatants were collected and stored at −70 to −80 °C until virus titration. Titration of virus was performed by standard plaque assay on Vero cell monolayers. Briefly, supernatant from homogenized tissues, serum, BAL, nasal flush, or AGI samples were serially diluted in EMEM ( Cellgro, Mediatech, Inc.) containing 5 % fetal bovine serum (FBS) (GIBCO Invitrogen Corp.), 1 % penicillin (20,000 IU/ml)-streptomycin sulfate (20,000 μg/ml), 1 % non-essential amino acids (NEAA) (Sigma Aldrich Company, Inc., St. Louis, MO), 1 % 200 mM L-glutamine (Thermo Scientific, Logan, UT), and 0.1 % gentamicin solution (Sigma Aldrich Company, Inc.). Diluted samples were then added in duplicate to 6-well plates containing confluent monolayer of Vero cells (African green monkey kidney cells) which were incubated at 37 °C for 1 hour, with rocking every 15 min. Following the incubation period, a 0.5 % agarose overlay in 2x EBME solution (GIBCO, Life Technologies) with HEPES and 10 % FBS, 1 % L-glutamine, 1 % NEAA, 1 % penicillin-streptomycin sulfate, and 0.1 % gentamicin was added, and plated were incubated at 37 °C at 5 % CO_2_ for 24 hr. Thereafter, a second agarose overlay in 2x EBME containing supplements and 5 % neutral red was added. The plates were again incubated at 37 °C at 5 % CO_2_ for 24 hr. Defined plaques (neutral red exclusion areas) were then counted. The limit of detection for this assay was 5 pfu/ml.

## Additional file

Additional file 1: Figure S1. Geometric mean viral titer in the liver, heart, kidney, adrenal gland, and pancreas of the groups from BALB/c mice infected with NA EEEV strain FL93-939 (n = 5). Viral titers of tissue homogenate supernatants were determined by standard plaque assay. The limit of detection of the assay is 5 pfu/ml tissue homogenate supernatant. (PPTX 64 kb)

## References

[1] Arrigo NC, Adams AP, Weaver SC (2010). Evolutionary patterns of eastern equine encephalitis virus in North versus South America suggest ecological differences and taxonomic revision. J Virol.

[2] Carrera JP, Forrester N, Wang E, Vittor AY, Haddow AD, Lopez-Verges S (2013). Eastern equine encephalitis in Latin America. N Engl J Med.

[3] Steele KE, Twenhafel NA (2010). REVIEW PAPER: pathology of animal models of alphavirus encephalitis. Vet Pathol.

[4] Aguilar PV, Paessler S, Carrara AS, Baron S, Poast J, Wang E (2005). Variation in interferon sensitivity and induction among strains of eastern equine encephalitis virus. J Virol.

[5] Gardner CL, Burke CW, Tesfay MZ, Glass PJ, Klimstra WB, Ryman KD (2008). Eastern and Venezuelan equine encephalitis viruses differ in their ability to infect dendritic cells and macrophages: impact of altered cell tropism on pathogenesis. J Virol.

[6] Gardner CL, Yin J, Burke CW, Klimstra WB, Ryman KD (2009). Type I interferon induction is correlated with attenuation of a South American eastern equine encephalitis virus strain in mice. Virology.

[7] Schneider BS, Soong L, Zeidner NS, Higgs S (2004). Aedes aegypti salivary gland extracts modulate anti-viral and TH1/TH2 cytokine responses to sindbis virus infection. Viral Immunol.

[8] Van den Broeck W, Derore A, Simoens P (2006). Anatomy and nomenclature of murine lymph nodes: Descriptive study and nomenclatory standardization in BALB/cAnNCrl mice. J Immunol Methods.

[9] Dyce KM, Sack WO, Wensing CJG (1987). Textbook of Veterinary Anatomy.

[10] Vogel P, Kell WM, Fritz DL, Parker MD, Shoepp RJ (2005). Early events in the pathogenesis of eastern equine encephalitis virus in mice. Am J Pathol.

[11] Gardner CL, Ebel GD, Ryman MD, Klimstra WB (2011). Heparin sulfate binding by natural eastern equine encephalitis viruses promotes neurovirulence. Proc Natl Acad Sci U S A.

[12] Reed DS, Lackemeyer MG, Garza NL, Norris S, Gamble S, Sullivan LJ (2007). Severe encephalitis in cynomolgus macaques exposed to aerosolized Eastern equine encephalitis virus. J Infect Dis.

[13] Adams AP, Aronson JF, Tardif SD, Patterson JL, Brasky KM, Geiger R (2008). Common marmosets (Callithrix jacchus) as a nonhuman primate model to assess the virulence of eastern equine encephalitis virus strains. J Virol.

[14] Roy CJ, Reed DS, Wilhelmsen CL, Hartings J, Norris S, Steele KE (2009). Pathogenesis of aerosolized Eastern Equine Encephalitis virus infection in guinea pigs. Virol J.

[15] Honnold SP, Mossel EC, Bakken RR, Lind CM, Cohen JW, Eccleston LT, et al. *Eastern equine encephalitis virus in mice II: Pathogenesis is dependent on route of exposure.* Virol J. Accepted for publication, 2015. 10.1186/s12985-015-0385-2 PMC4589026 26423229

[16] Calisher CH (1994). Medically important arboviruses of the United States and Canada. Clin Microbiol Rev.

[17] Guyton AC (1947). Measurement of the respiratory volumes of laboratory animals. Am J Physiol.

