# Supplementary figures and images for: Eastern equine encephalitis virus in mice I: clinical course and outcome are dependent on route of exposure
Source: Virol J. 2015 Sep 29;12:152. doi: 10.1186/s12985-015-0386-1 (PMC4588493; doi:10.1186/s12985-015-0386-1)

## Slide 1
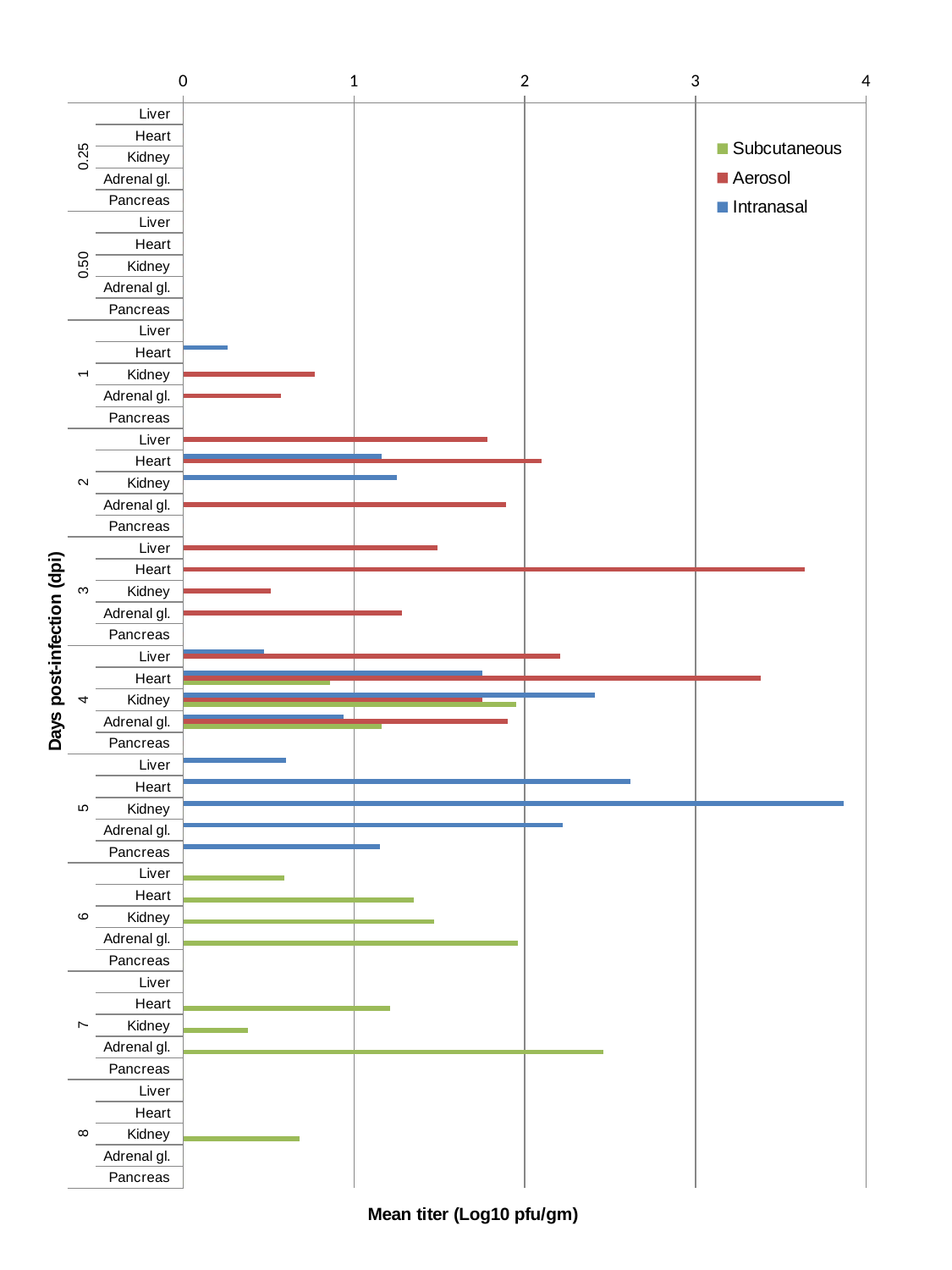

[unsupported chart]

Supplement: Additional file 1: Figure S1. — Geometric mean viral titer in the liver, heart, kidney, adrenal gland, and pancreas of the groups from BALB/c mice infected with NA EEEV strain FL93-939 (n = 5). Viral titers of tissue homogenate supernatants were determined by standard plaque assay. The limit of detection of the assay is 5 pfu/ml tissue homogenate supernatant. (PPTX 64 kb) [file 12985_2015_386_MOESM1_ESM.pptx]
